# Simultaneous Repair of Pseudo Para‐Anastomotic Aneurysm and Abdominal Aortic Aneurysm With Hybrid Approach

**DOI:** 10.1002/ccr3.70999

**Published:** 2025-09-24

**Authors:** Hiroki Moriuchi, Nobuhiro Shimabukuro, Akihiko Yamauchi

**Affiliations:** ^1^ Department of Cardiovascular Surgery Yuuai Medical Center Okinawa Japan

**Keywords:** abdominal aortic aneurysm, hybrid approach, para‐anastomotic aneurysm, vascular surgery

## Abstract

Para‐anastomotic aneurysm (PAA) is a rare and severe complication after open surgery for aortic aneurysm or occlusive disease. A 65‐year‐old man with a history of left common iliac‐femoral artery bypass surgeries for occlusive disease 15 years ago presented with a femoral pseudo PAA and AAA. Endovascular repair and surgical clamping of the proximal artery of the pseudo PAA were considered technically challenging due to complex anatomy and severe adhesions. We used an occlusion balloon to control bleeding from the pseudo PAA and performed surgical repair of the pseudo PAA and AAA simultaneously. Simultaneous repair of pseudo PAA and AAA with a hybrid approach is extremely rare. Although the number of endovascular aneurysm repair cases has increased, some cases, like this one, still require open surgery.

**Summary**

- Simultaneous repair of a pseudo PAA and an abdominal aortic aneurysm is extremely rare.
- A hybrid approach is a highly effective method for managing such cases.

## 1 Introduction

Para‐anastomotic aneurysm (PAA) is a major complication following open surgery for aortic aneurysm or occlusive disease. The incidence of PAA is low in the short term but increases significantly over time after the initial operation [1, 2]. Repair of PAA is challenging due to the severe adhesions surrounding the aneurysm, which increase the risk of bleeding and injury to adjacent organs. This report is a rare and educational case of simultaneous repair of a femoral pseudo PAA and an abdominal aortic aneurysm (AAA) using a hybrid approach. Written informed consent was obtained from the patient.

## 2 Case History

A 65‐year‐old man was referred to our hospital with left inguinal swelling and pain. He had undergone left common iliac artery to left common femoral artery bypass surgery for left CIA occlusion 15 years ago. Subsequently, due to early graft occlusion, a femoral‐femoral (F‐F) bypass was performed. The patient did not have a fever but complained of significant swelling and pain in the groin area.

## 3 Investigations and Treatment

**FIGURE 1:**
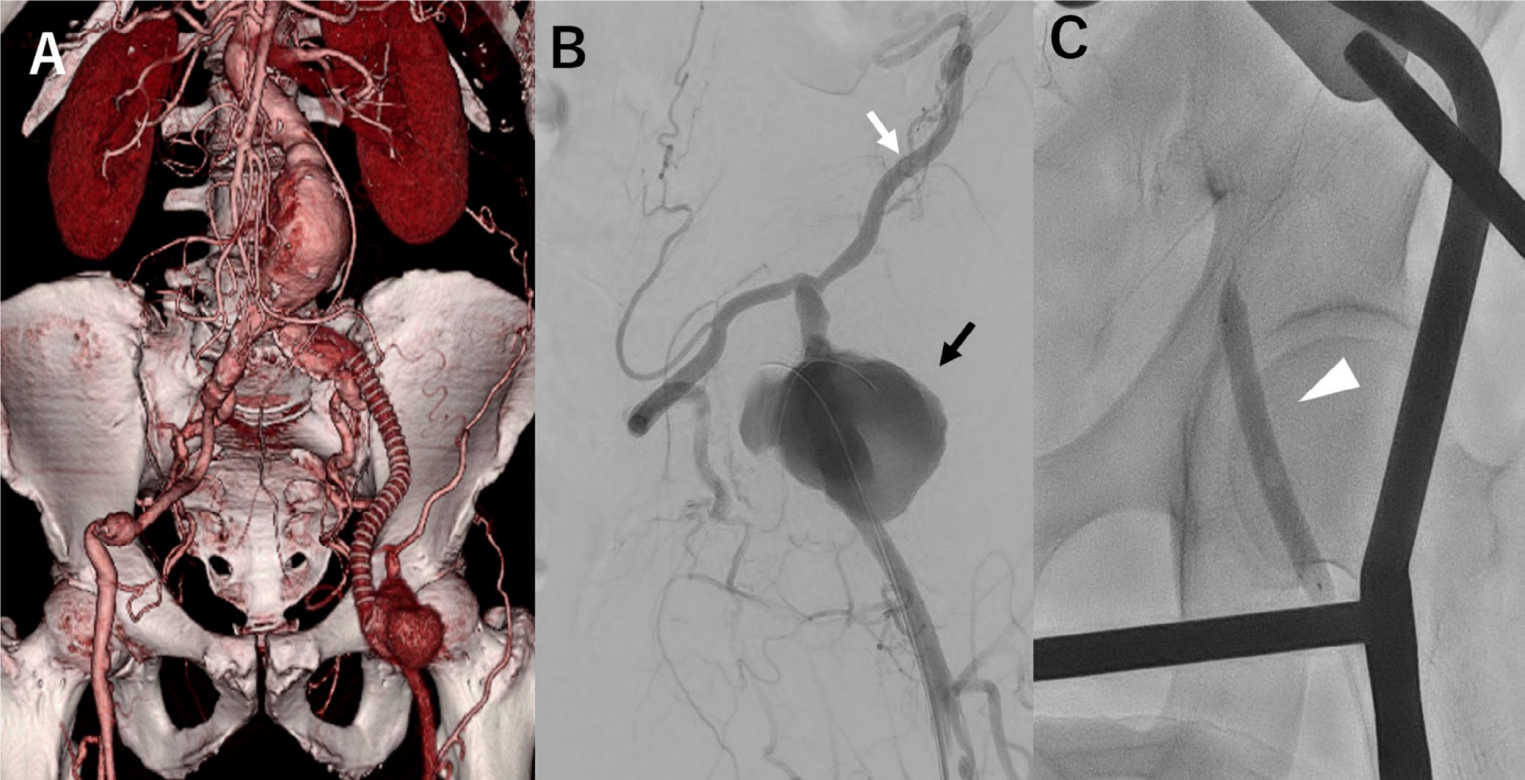
(A) Preoperative computed tomography showed abdominal aortic aneurysm and left femoral pseudo aneurysm. (B) Angiography revealed femoral pseudo aneurysm (black arrow) and circumflex iliac artery (white arrow). (C) Occlusion balloon was placed at common femoral artery (white arrow head).

Contrast‐enhanced computed tomography revealed a large pseudoaneurysm at the left common femoral artery (60 × 55 mm) and an abdominal aortic aneurysm (65 × 68 mm). The left CIA‐to‐left CFA graft and the right CFA‐to‐left CFA graft were occluded, with detachment at the distal anastomosis resulting in a pseudoaneurysm (Figure 1A). Both aneurysms were large and caused the patient pain; therefore, emergency surgery was performed.

Under general anesthesia with the patient in the supine position, a longitudinal incision was made in the left thigh. We exposed the left superficial femoral artery and inserted a 6Fr sheath to CFA. A 0.018 guidewire (TERUMO Co., Tokyo, Japan) was passed through the circumflex iliac artery to prepare balloon occlusion. The abdominal aortic aneurysm (AAA) was exposed transperitoneally through a median abdominal incision. After heparinization, the infrarenal aorta and bilateral CIA were clamped, and the sac was opened. The 18 × 9 mm J graft (Lifeline, Tokyo, Japan) was end to end anastomosed to the infrarenal aorta and right CIA. A 7 mm × 6 cm SABERX (Cordis, Cardinal Health, OH, USA) was inserted into the left CFA and inflated to control the bleeding (Figure 1B,C). Following balloon occlusion, the pseudoaneurysm was opened, and bleeding from the side branches was controlled. With a clear surgical field achieved, we removed the occlusion balloon and clamped the distal CFA. The J graft was end to end anastomosed to the left CFA. After confirming good blood flow to the extremities and hemostasis, the procedure was completed.

## 4 Outcome and Follow‐Up

**FIGURE 2:**
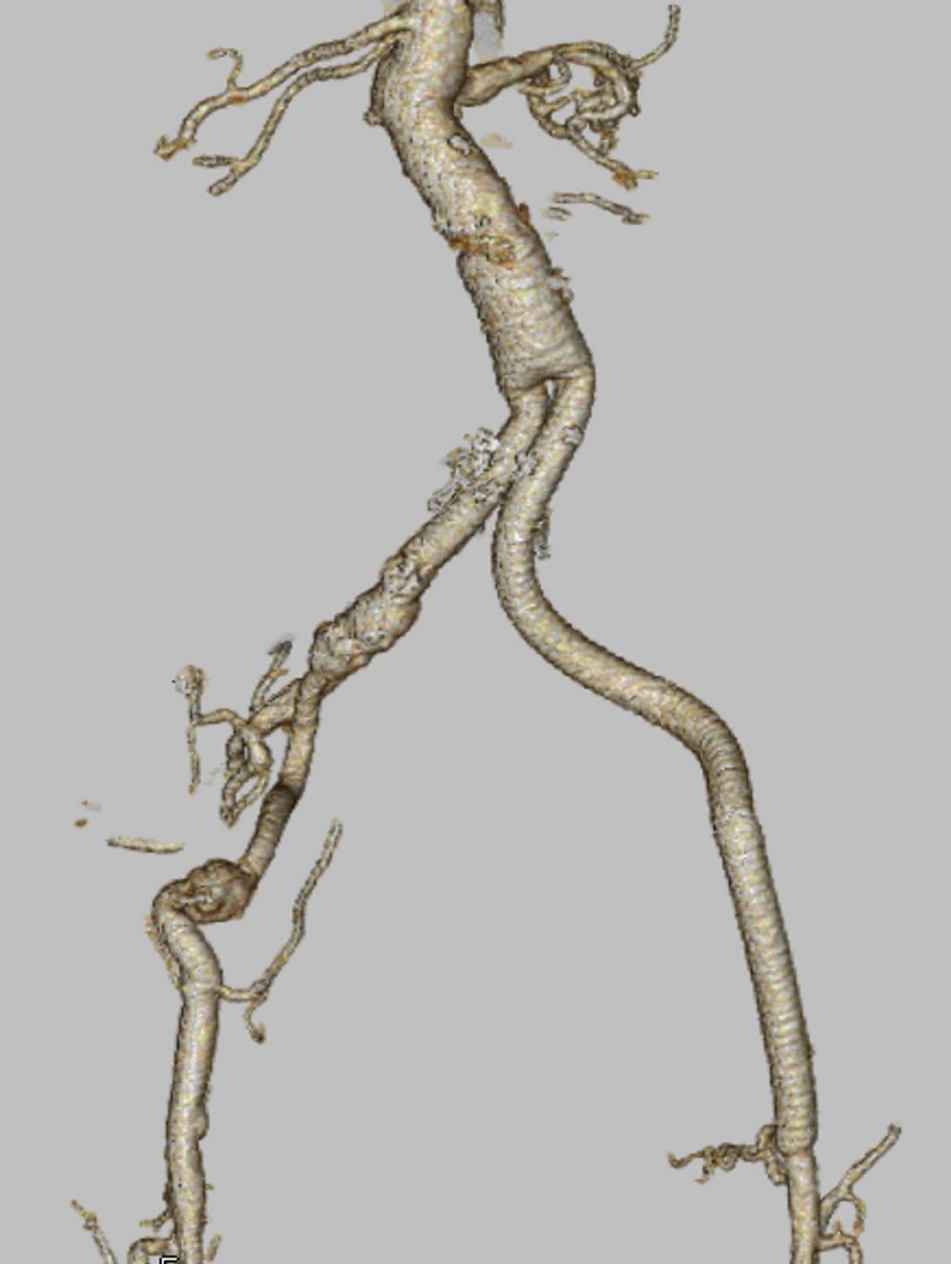
Postoperative computed tomography. 3D image showed good anastomosis and no aneurysm.

Postoperative course was uneventful, CT revealed no anastomotic aneurysm and no stenosis of graft (Figure 2). Intraoperative cultures were negative, and the absence of fever along with a low inflammatory response in the patient ruled out infection. He was discharged after 10 days from the operation.

## 5 Discussion

PAA is a severe complication after open surgery for aortic aneurysm or occlusive disease. The overall incidence of PAA has been reported to range from 0.2% to 15% [1]. Technical errors, major perioperative complications, recurrent aneurysms, and α1‐antitrypsin deficiency may contribute to the early onset of pseudoaneurysm [3].

The operative risk of PAA is higher than primary grafting because of severe adhesion, the risk of injury to surrounding tissue, older age, and the need for higher aortic clamp. Surgical repair is the standard of treatment of PAA, but endovascular therapy has been reported to reduce perioperative mortality and morbidity, but anatomical suitability is necessary.

In the present case, the patient presented with pain, and the size of the both aneurysms was so large that urgent surgery was performed. Endovascular treatment is challenging due to the patient's previous left CIA‐CFA bypass surgery for left CIA occlusion. Although aorto‐uni‐iliac device replacement and femoral‐femoral bypass surgery are required, the procedure is technically difficult because of the prior surgeries and the presence of a pseudo PAA. Therefore, we performed open surgery with a hybrid approach, using an occlusion balloon to control bleeding from the proximal artery of the femoral PAA. In fact, clamping without bleeding was highly challenging due to severe adhesion in the surrounding tissue of the pseudo PAA. We successfully occluded the femoral artery with an occlusion balloon; the pseudo PAA was opened without major bleeding. The left CIA was occluded, but balloon blockade was necessary to control blood flow from collateral vessels.

When endovascular repair is selected, intraoperative troubleshooting and long‐term follow‐up are critically important [4, 5, 6]. Recently, although the number of endovascular aneurysm repair cases has increased, some cases, like the present one, still require open surgery.

## 6 Conclusion

Simultaneous surgical repair of femoral pseudo PAA and AAA with a hybrid approach is extremely rare. This method is very effective in this case, but long‐term follow‐up is necessary.

## Author Contributions

**Hiroki Moriuchi:** conceptualization, data curation. **Nobuhiro Shimabukuro:** conceptualization. **Akihiko Yamauchi:** conceptualization.

## Ethics Statement

The patient's consent was obtained.

## Conflicts of Interest

The authors declare no conflicts of interest.

## Data Availability

All relevant data is within the manuscript.
